# Obesity and dietary fat influence dopamine neurotransmission: exploring the convergence of metabolic state, physiological stress, and inflammation on dopaminergic control of food intake

**DOI:** 10.1017/S0954422421000196

**Published:** 2021-06-28

**Authors:** Conner W. Wallace, Steve C. Fordahl

**Affiliations:** The Department of Nutrition, UNC Greensboro, Greensboro, NC 27412, USA

**Keywords:** High-fat diet, Dopamine neurotransmission, Nucleus accumbens, Homeostatic feeding, Hedonic feeding, Inflammation, Insulin resistance, Diet-induced obesity, Hypothalamic–pituitary–adrenal stress axis, Kappa-opioid receptors

## Abstract

The aim of this review is to explore how metabolic changes induced by diets high in saturated fat (HFD) affect nucleus accumbens (NAc) dopamine neurotransmission and food intake, and to explore how stress and inflammation influence this process. Recent evidence linked diet-induced obesity and HFD with reduced dopamine release and reuptake. Altered dopamine neurotransmission could disrupt satiety circuits between NAc dopamine terminals and projections to the hypothalamus. The NAc directs learning and motivated behaviours based on homeostatic needs and psychological states. Therefore, impaired dopaminergic responses to palatable food could contribute to weight gain by disrupting responses to food cues or stress, which impacts type and quantity of food consumed. Specifically, saturated fat promotes neuronal resistance to anorectic hormones and activation of immune cells that release proinflammatory cytokines. Insulin has been shown to regulate dopamine neurotransmission by enhancing satiety, but less is known about effects of diet-induced stress. Therefore, changes to dopamine signalling due to HFD warrant further examination to characterise crosstalk of cytokines with endocrine and neurotransmitter signals. A HFD promotes a proinflammatory environment that may disrupt neuronal endocrine function and dopamine signalling that could be exacerbated by the hypothalamic–pituitary–adrenal and κ-opioid receptor stress systems. Together, these adaptive changes may dysregulate eating by changing NAc dopamine during hedonic versus homeostatic food intake. This could drive palatable food cravings during energy restriction and hinder weight loss. Understanding links between HFD and dopamine neurotransmission will inform treatment strategies for diet-induced obesity and identify molecular candidates for targeted therapeutics.

## Introduction

Overweight and obesity prevalence has steadily increased with 42·4 % of US adults currently classified as obese^1^. Food intake is controlled by many factors, including an obesogenic food environment with ubiquitous access to cheap, calorie-rich, palatable foods. Herein, ‘palatable foods’ are defined as those with high energy density primarily from fat or sugar. Over-consumption of palatable food is proposed to shift brain dopamine signalling within the nucleus accumbens (NAc)^2,3^. The NAc is a limbic–motor interface which integrates salient stimuli with memory and context, reward availability and value, sensory information, physiological state and homeostatic needs^4^. It sends efferent projections to cortical and motor regions, processing environmental and biological stimuli to drive motivated behaviour. Disrupting homeostasis in the NAc by stimulating excessive dopamine release may contribute to obesity with extended access to highly palatable foods that acutely cause phasic dopamine release in the NAc^5,6^. Perturbations to NAc dopamine by consuming a diet high in saturated fat (HFD) may disrupt natural NAc food reward learning and reduce NAc dopamine tone over time^7–11^. Changes in dopamine tone or phasic dopamine release in the NAc may significantly impact food seeking^8,11–13^, reward^8,9,11,12^ and satiety^11,13,14^. However, the mechanisms by which diet and obesity alter dopamine neurotransmission and behaviour are not fully characterised. Therefore, the purpose of this review is to highlight the literature and identify research gaps related to mechanisms by which diet-induced obesity interfere with NAc dopamine, including interactions between inflammation, physiological stress and κ-opioid receptor function which together with endocrine hormones modulate NAc dopamine to influence food intake behaviours.

## Dopamine circuitry and motivated behaviour

**Fig. 1.**
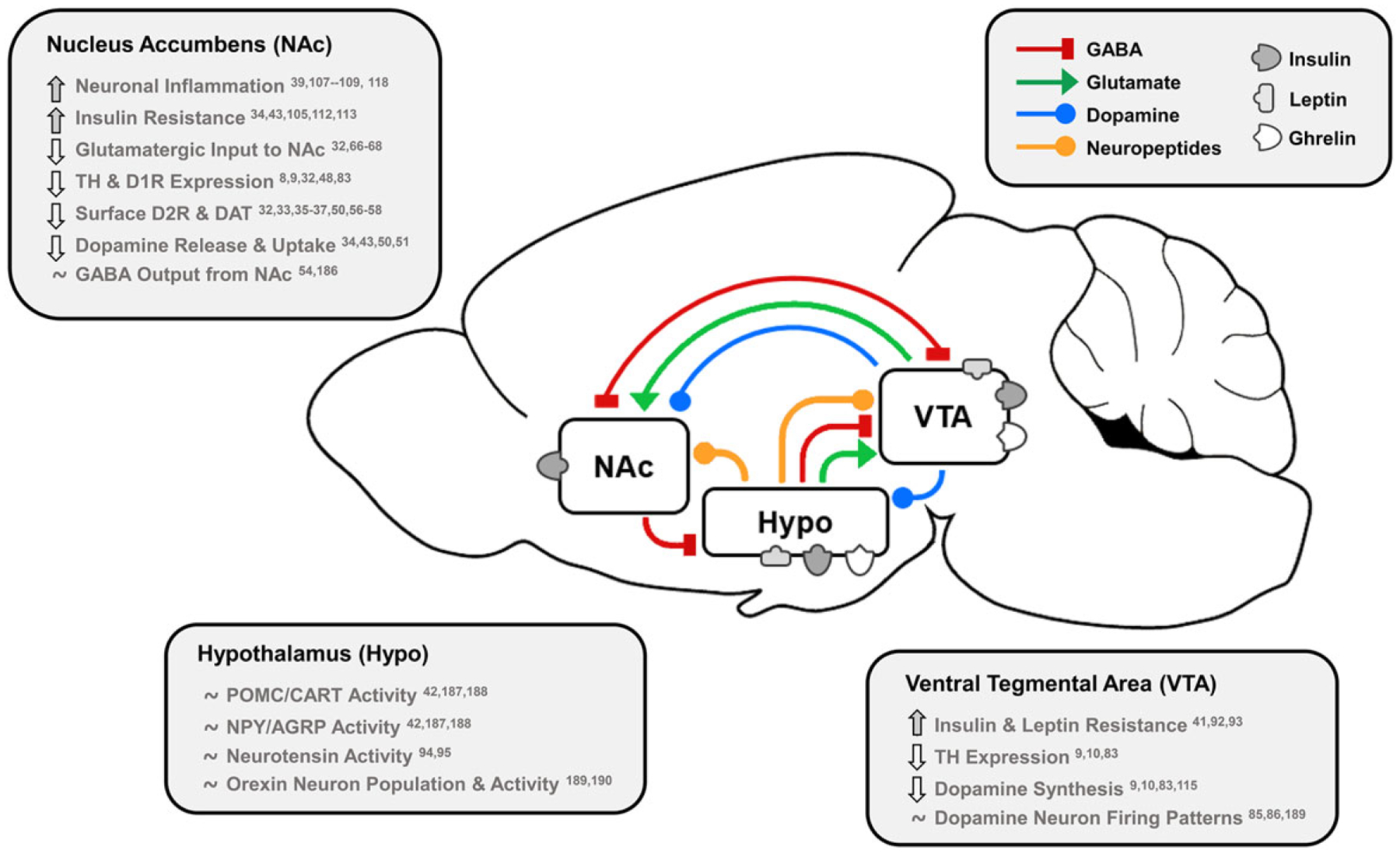
Effects of dietary fat and obesity on hedonic and homeostatic dopamine circuits: homeostatic, dopamine-motivated feeding and reward learning circuits overlap as insulin and leptin convey body energy status to the hypothalamus (Hypo) and VTA. In response, hypothalamic nuclei send appetitive neuropeptides to the VTA and NAc to influence food intake, and NAc dopamine neurotransmission is directly stimulated by hormonal action in the NAc and VTA. This information is also conveyed via dopamine, GABA and glutamate from the VTA to NAc, and the NAc responds by sending GABA to hypothalamic feeding regions, the VTA as a regulatory feedback circuit, and thalamic, motor and cognitive cortical regions. Effects of long-term HFD or palatable food consumption are highlighted by region. This characterises how diet-induced obesity dysregulates key neurotransmitters, neuropeptides and hormones that regulate food intake to reduce dopamine neurotransmission leading to overeating and further weight gain. TH, tyrosine hydroxylase; D1R/D2R, dopamine type 1/2 receptors; DAT, dopamine transporter; POMC/CART, pro-opiomelanocortin/cocaine- and amphetamine-regulated transcript; NPY/AGRP, neuropeptide Y/agouti-related peptide.

Food intake is controlled by energy status and neural circuits regulating homeostasis and reward. Two primary dopamine circuits include the nigrostriatal tract from substantia nigra to dorsal striatum/caudate putamen controlling motivation and habitual behaviour, and the mesocorticolimbic tract from ventral tegmental area (VTA) to NAc controlling Pavlovian reward learning^15^. These dopamine neurons form synaptic terminals with γ-aminobutyric (GABA)-releasing medium spiny neurons (MSNs), comprising ~90–95 % of neurons within the NAc^16^, which express dopamine D1 receptor (D1R) and dopamine D2 receptor (D2R) subtypes that propagate dopamine signalling to control cortical and motor processing^17^. An excellent review of homeostatic crosstalk with the dopamine reward system by Ferrario and colleagues highlighted how glucose and endocrine indicators of energy status (insulin, leptin) inhibit or excite dopaminergic reward activity directly in the VTA, NAc and striatum^2^. Hormones also indirectly modulate dopaminergic activity by targeting key homeostatic regions in the hypothalamus that initiate food seeking behaviours via GABAergic and glutamatergic inputs to VTA and NAc^2,18^. There are multiple subtypes of these neurons within the lateral hypothalamus (LH) and arcuate nucleus (ARC) that respond to energy status by releasing appetitive neuropeptides. These include orexin/hypocretin (orexigenic, LH to VTA/NAc), melanin-concentrating hormone (orexigenic, LH to NAc), neurotensin (anorectic, LH to VTA/LH orexin neurons), neuropeptide Y (NPY)/agouti-related peptide (AGRP) (orexigenic, ARC to LH) and pro-opiomelanocortin (POMC)/cocaine- and amphetamine-regulated transcript (CART) (anorectic, ARC to VTA/NAc)^2^. These appetitive systems underly many mechanisms by which HFD and stress perturb dopamine control of food intake and will be discussed in detail throughout this review. In addition to homeostatic engagement of hypothalamus afferents to the VTA and NAc, feeding is also controlled by a NAc to hypothalamus satiety circuit. This was demonstrated by direct inhibition or stimulation of D1R-expressing MSNs that project from the NAc shell to the LH, where D1R inhibition increased licking for fat and sugar but stimulation decreased ingestive responses^19^. Overall, NAc dopamine neurotransmission and subsequent GABA output controls motivated behaviour, and homeostatic signals from the hypothalamus comprise important inputs that regulate feeding. Therefore, diet-induced disruption to these circuits may be particularly consequential for individuals restricting food intake (Fig. 1).

The ventral striatum is central to reward processing, integrating glutamatergic and GABAergic inputs from the hypothalamus, cortex, amygdala and hippocampus with dopaminergic projections from the VTA or substantia nigra^20^. The NAc assimilates these signals to determine hedonic value and sends GABA via the ‘direct’ route with direct control of dopamine release with afferents to VTA, internal globus pallidus or substantia nigra (MSNs with D1Rs), or the ‘indirect’ route via globus pallidus externa and ventral pallidum (MSNs with D2Rs). GABA afferents to the thalamus then modulate excitatory output to the cortex that controls behavioural selection and motor activity^17^. D1Rs have lower affinity for dopamine than do D2Rs^21,22^ and respond to phasic dopamine release due to unexpected rewards and cue learning that promotes cyclic adenosine monophosphate (cAMP) signalling with downstream phosphorylation of dopamine-regulating proteins^15^ and increased MSN firing probability^23^. Conversely, D2Rs are activated at lower dopamine concentrations by spontaneous pacemaking activity of dopamine neurons^24^ than D1Rs, and activate opposing intracellular signalling, to decrease MSN firing probability^23^. Consequently, D2Rs communicate dopamine tone so that phasic release events (unexpected rewards) or lack thereof (absence of expected reward) alter concentration that is detected by D1Rs to promote response. This dopamine signalling pattern is important for NAc learning that relies on phasic dopamine release initiated by a rewarding, unconditioned stimulus that becomes tied to a conditioned cue over repeated exposures. In the theory of reward prediction error^25^, hedonic value is determined based on magnitude of dopamine release, which is up- or down-regulated upon further cue exposures. This reward-learning model is central to motivation and survival and may become ‘hijacked’ by palatable foods.

## Dopamine in the NAc responds to food intake and may promote obesity

Dopamine pathways control motivated and habitual behaviour, including that related to food. The VTA–NAc mesolimbic dopaminergic pathway influences motivated behaviour by enhancing willingness to work for rewards^26^. Mesolimbic dopamine increased during lever pressing to obtain food^27^, and NAc core dopamine depletion reduced response in fixed ratio tasks with more pronounced decreases in higher ratio schedules^28^. Moreover, food deprivation augments dopaminergic responses to food, demonstrated by increased NAc dopamine in response to maize oil feeding after food restriction^6^. Interestingly, phasic dopamine release was enhanced in food-restricted rats following cues that predict sucrose versus cues for saccharin^5^. This suggested energy-providing foods have greater salience than energy-null foods when homeostatic energy needs influence physiological state, but dopamine release induced by food cues that initiate food intake could be enhanced in individuals with obesity. Indeed, those who were obese showed enhanced striatal and NAc dopamine release in response to palatable food images after consumption of an energy-dense meal^29^. This was in contrast to healthy, lean individuals whose meal pleasantness ratings correlated with striatal dopamine release^30^ and who experienced striatal dopaminergic activation immediately during milkshake consumption and 20 min post-ingestion when gut signals reached the brain^31^. Additionally, when food access is not interrupted but availability of palatable foods is limited, dopamine release is increased during access to the preferred food under limited compared with *ad libitum* access in rodents. For instance, constant access to sucrose for 21 d failed to evoke the same magnitude of dopamine release in the NAc shell as daily intermittent access to sucrose^14^. Therefore, dopamine release in response to food can be influenced by physiological state and food availability, and the NAc controls initial hedonic responses to palatable food intake and promotes cue-associated learning and motivation to obtain food that is interfaced with homeostatic need.

## Effects of HFD on dopamine

Prolonged consumption of highly palatable diets may disrupt dopamine reward signalling. Chronic HFD intake and diet-induced obesity impact VTA dopamine neuron activity and interfere with mechanisms regulating dopamine at synaptic terminals within the NAc. Changes include lowered D2R binding potential as well as reduced dopamine transporter (DAT) function and membrane localisation^32–37^. Furthermore, HFD intake activates inflammatory processes that may contribute to neuronal insulin resistance^34,38–40^. Central insulin and leptin resistance attenuate satiation and reward valuation of palatable foods by altering NAc synaptic dopamine and disrupt orexigenic and anorectic communication between the LH, ARC and VTA^34,40–43^. Finally, chronic HFD intake shifts opioid control of NAc dopamine neurotransmission^44^, which could amplify stress-induced feeding^8,45^ and have consequences for obese individuals on energy restricted diets. Overall, studies presented below demonstrate HFD consumption acutely increases NAc dopamine^6,46,47^, but prolonged intake reduces capacity for dopamine neurotransmission through repeated stimulation of dopamine receptors^9,48^, resistance to hormonal and homeostatic signals^34,40–43^, and up-regulated inflammatory signalling^38,39^.

### Effects on synaptic control of dopamine within the NAc

#### Dopamine transporter, D1 receptors and D2 receptor availability.

Chronic HFD intake alters dopamine neurotransmission to promote food seeking with obesity susceptibility dependent on the activation of NAc dopamine and alteration to dopamine receptors and the DAT. For example, genetic differences in the DAT gene significantly increased likelihood of obesity^49^. Further, intake of a HFD or intraperitoneal injection of lipid solution acutely increased NAc dopamine^46^, and sucrose intake dose-dependently increased NAc dopamine^47^. Conversely, chronic HFD feeding reduced maximal dopamine reuptake rate (*V*_max_)^34^, while extended access to a Western diet (WD), a HFD with added sugar, decreased striatal and NAc core dopamine release and reuptake^43^. Further, while obesogenic diet and food restriction both reduced striatal DAT surface expression and reuptake^50^, obesogenic diet decreased but food restriction increased D2R protein expression. These findings suggest food restriction primes the dopamine system to respond to food exposure. HFD-induced changes develop over time, as 6-week but not 2-week exposure to HFD decreased NAc dopamine *V*_max_ and membrane-associated DAT expression^33^. Effects of HFD also depend on fat type, as rats chronically fed 50 % saturated HFD experienced reduced DAT and increased D1R protein expression versus 50 % monounsaturated olive oil or control diet^32^. Similarly, consumption of a HFD versus control diet reduced dopamine reuptake *V*_max_ and attenuated phasic dopamine release, which did not occur in mice fed a diet high in polyunsaturated flax-seed oil^51^. Further, consumption of a WD versus low-fat control diet for 12 weeks reduced NAc D1R protein expression but increased total D2R and p-dopamine- and cAMP-regulated phosphoprotein-32 (DARPP-32) protein expression^9^, the latter which activates D1R-mediated signalling downstream^52^. HFD feeding from lactation through adulthood similarly increased NAc DARPP-32 but decreased D1R and D2R gene expression that was exacerbated after HFD was removed for 4 weeks^48^. Conversely, HFD-induced reduction in D1Rs and D2Rs was restored by HFD removal. These results suggest adaptive changes in DAT, D1R and D2R expression due to repeated HFD-mediated activation of D1R signal transduction leading to down-regulated D1R and D2R availability. Interestingly, D1R-expressing MSNs were activated in proportion to palatable food consumption while intra-uterine protein-restriction-induced reduction of NAc shell D1Rs increased palatable milk consumption^53^, leading Durst *et al*. (2019) to suggest D1R stimulation during consumption builds to a ‘satiety threshold’ sent to LH GABA neurons. Endocannabinoid-mediated synaptic plasticity of this circuit was later demonstrated to promote overeating after food restriction or exposure to HFD^54^. Therefore, the NAc–LH satiety circuit may be inhibited due to HFD-induced disruption of D1R signalling.

Much attention has also been paid by neuroimaging studies to striatal D2R availability. Decreased NAc core but increased NAc shell D2R binding potential were linked to impulsive behaviour^55^. Furthermore, calorie restriction was used to maintain similar intake between groups, and chronic HFD in absence of obesity decreased NAc D2R protein expression and increased impulsivity in a task to obtain food^56^. Conversely, obesity in absence of diet manipulations also affects D2Rs, as chow-fed, obese mice with genetic leptin receptor inactivation increased NAc and striatal D2R availability *in vivo* using [^11^C]raclopride but decreased D2R availability *ex vivo* using [^3^H]spiperone^57^. Interestingly, these differences were eliminated between calorie-restricted lean and obese mice^57^. Based on the radio-ligands used, results suggested obesity reduced striatal dopamine concentration and postsynaptic D2R availability. Conversely, 3–4 weeks of limited daily access to a cafeteria diet reduced ethanol intake but increased D2R autoreceptor function^58^. One group used [^3^H]raclopride^35–37^ to assess diet-induced alterations to D2Rs over time and found that 20 d on a 40 % HFD versus chow increased D2R binding density in the NAc and striatum that was maintained after HFD removal^35^. Conversely, diet-induced obesity that developed over 20 weeks of HFD feeding decreased striatal D2R binding^36^ but increased NAc core and striatal D2R mRNA expression^37^. Further, obesity-resistant mice had increased NAc DAT binding^36^, which may promote dopamine clearance and protect against obesity. These studies suggest palatable foods up-regulate dopamine neurotransmission which is shifted by chronic HFD intake to decreased D2R binding and capacity for dopamine release. However, clinical research showed negative correlation between age and D2R binding throughout midbrain regions with body mass index (BMI) correlating positively with D2R availability only for those over 30 years old^59^. This suggests that adolescent striatal development and decline of D2R expression patterns with age could partially explain associations between D2R availability and BMI. Overall, HFD-induced obesity alters function and expression of NAc proteins regulating dopamine to reduce capacity for NAc dopaminergic reward that promotes overeating and weight gain.

#### Acetylcholine, GABA and glutamate in the NAc.

Control of VTA–NAc dopamine signals that initiate motivated actions relies on a complex network of acetylcholine, glutamate and GABA, which are all affected by HFD intake. About 5 % of NAc neurons are GABAergic or cholinergic interneurons (CIN)^17^. The latter stimulate dopamine release via acetylcholine that activates acetylcholine receptors on dopamine axon terminals^60^. Activation of MSNs relies on glutamate targeting ionotropic *N*-methyl-d-aspartate (NMDA) and α-amino-3-hydroxy-5-methyl-4-isoxazole propionic acid receptors (AMPAR) on CINs to release acetylcholine^60,61^. Glutamate also negatively regulates dopamine directly by metabotropic glutamate receptors^62^ and indirectly via MSN retrograde H_2_O_2_ release^63^. Glutamatergic inputs to NAc communicate physical and nutritive qualities of food, memory, physiological need and environmental cues^20^, which prompts initiation or cessation of feeding. Indeed, either AMPA and NMDA receptor agonism^64^ or antagonism^65^ in the NAc shell can induce voracious feeding. However, consumption of a WD^66^ or a HFD^32,67,68^ increased AMPA/NMDA receptor ratio^66,67^, prolonged excitatory postsynaptic currents onto MSNs^66^, and increased NAc phosphorylated GluR1 AMPA subunit^32^ and NAc shell NMDAR and metabotropic glutamate receptor^68^ expression, but inhibited ability to induce long-term depression onto MSNs^67^, effects which together increased motivation to obtain palatable food^67,68^. Further, the NAc receives GABA from VTA^69^, globus pallidus externa^70^, cortex^71^, bed nucleus of the stria terminalis^72^, and local MSNs and interneurons^24^. GABA signals reduce dopamine concentration^73^ to stop cue-associated reward behaviour^70^ or food intake and induce avoidance behaviour^69,71^. These effects occur directly via dopamine axon terminal^74^ GABA_B_-receptor-mediated reduction of dopamine^73^ and indirectly via GABA_A_-receptor-mediated reduction of acetylcholine^69,73^. Conversely, NAc shell GABA_A_ and GABA_B_ antagonism decreased food intake while fasted^75^, but agonism increased fat and sucrose^76^ as well as regular food intake while sated^77^, suggesting GABA inhibits NAc-LH MSNs to disinhibit feeding. However, GABA_B_ agonism also inhibited bingeing on HFD during an intermittent access paradigm^78^. Overall, glutamatergic and cholinergic signalling within NAc promotes food intake, while the system of GABAergic disinhibition throughout the NAc, VTA and hypothalamus may decrease dopamine release and stop intake or disinhibit feeding via NAc shell MSNs, but prolonged HFD intake reduces sensitivity of these systems to promote overeating and highlights the complexity of dopaminergic regulation of ingestive behaviours.

### Effects on the VTA

VTA dopaminergic output underlies motivated behaviours and is affected by consumption of a HFD^24,79,80^. The VTA receives glutamatergic and GABAergic signals from local interneurons and limbic and cortical regions^80^ as well as local somatodendritic dopamine release that negatively regulates dopamine neuron excitability^24^. Glutamatergic and cholinergic inputs activate receptors on VTA dopamine neurons to promote NAc dopamine release^81^. The VTA receives orexin from LH, NPY from orexigenic glucose-sensing ARC neurons^2,82^, and anorectic POMC/CART signals from ARC^2^. The VTA integrates this information related to homeostasis and environmental cues with dopamine neurons projecting to the NAc. However, HFD intake reduced VTA tyrosine hydroxylase (TH) mRNA^10^ and protein^9,83^ expression, which occurred regardless of obesity and was restored after switching to low-fat diet^10^. This showed HFD intake reduced capacity for dopamine synthesis, because TH is the rate-limiting enzyme in synthesising dopamine^84^. Further, 6-week *ad libitum* HFD feeding attenuated D2R agonist quinpirole-induced inhibition of VTA dopamine neuron firing, suggesting D2R desensitisation^85^, whereas intermittent access to cafeteria diet for 3 weeks increased inhibitory effects of quinpirole and reduced ethanol and sucrose intake^58^, showing sensitivity of VTA autoreceptor function to type and length of diet. HFD intake similarly reduced excitability of mouse VTA GABA neurons^86^ that reduce NAc dopamine concentration^69,73^ and stop behaviour (e.g. food intake)^69–71^. Overall, HFD and obesity affect VTA dopamine and GABA neuron protein expression and function, and a further diet-induced effect includes altered sensitivity to direct responses of VTA neurons to hormonal indicators of energy status.

VTA neurons express receptors for insulin and leptin^87^ with leptin-receptor-expressing dopamine and GABA neurons projecting to the NAc^88^, and activation of these receptors decreases food intake. Insulin in the VTA is important in reducing dopamine neuron activity to control behaviour, demonstrated by reduced locomotion and NAc dopamine after VTA application of insulin^89^. In the VTA, insulin reduced somatodendritic dopamine release and hedonic feeding, which was abolished by blocking the DAT^90^. Furthermore, insulin induced AMPAR- and endocannabinoid-mediated long-term depression in VTA dopamine neurons which was attenuated by genetic or diet-induced elevation of insulin^91,92^, showing a role of insulin in the VTA to signal satiety that is inhibited by HFD. VTA signalling is also affected by leptin resistance, as HFD consumption reduced ability of VTA leptin administration to limit food intake and weight gain in obesity-prone rats^93^ and induced leptin resistance specifically in the VTA and ARC^41^. Diet-induced obesity also spurred leptin resistance in ARC NPY/AGRP and POMC/CART neurons^42^ and LH neurotensin–galanin–GABA neurons^94,95^ necessary for reducing food intake by inhibiting orexin and activating VTA neurons^2,94,95^. Therefore, lack of hypothalamic and VTA insulin and leptin signalling may reduce capacity for NAc dopamine release to promote compensatory over-seeking of palatable food.

### Effects on anorectic hormones in the NAc

Insulin gains access to the brain via transport across the blood–brain barrier^96^ and local production in the brain^97^, and activation of tyrosine kinase receptors by insulin promotes phosphatidylinositol-3 kinase (PI3K) and protein kinase B (Akt) or the mitogen-activated protein kinase (MAPK)/extracellular-signal-regulated kinase (ERK) pathways, the latter which is also known as the Ras-Raf-MAPK/ERK kinase (MEK)-ERK cascade^96^. Insulin valuates food reward by fine-tuning dopamine neurotransmission at NAc dopamine terminals. For example, NAc core and shell insulin administration increased dopamine release and reuptake in control animals but not in animals consuming HFD^34,40^. Furthermore, HFD-induced impairments were reversed by promoting insulin receptor substrates, while effects of insulin were abolished by inhibiting insulin receptor or PI3K^34^, implicating HFD-induced insulin resistance in impaired dopamine control. Insulin receptors expressed on NAc dopamine neuron terminals^40^ activate Akt and ERK to shuttle DAT to the plasma membrane to promote dopamine reuptake^98^. Dopamine release is also promoted by insulin as NAc CINs expressed insulin receptors at high density^40^ and released acetylcholine in response to insulin^40,43^, supporting necessity of insulin in encoding sucrose preference^40^. While an obesogenic diet blunted insulin-induced NAc dopamine release and reuptake^43^, food restriction alternately enhanced insulin receptor expression and stimulated dopamine reuptake^50^. Similar effects have been shown with impaired leptin signalling, as leptin-deficient mice had reduced electrically evoked NAc shell dopamine release and reduced TH and DAT expression^88^, whereas leptin increased activity of NAc DAT and TH and increased amphetamine-evoked dopamine release^99^. Leptin activates intracellular signalling cascades similarly to insulin in addition to the Janus-activated kinases (JAK)–signal transducers and activators of transcription (STAT)–suppressors of cytokine signalling (SOCS) pathway that reduce NPY/AGRP but increase POMC/CART and LH neurotensin neuronal activity^2,100^. Further, leptin is also transported into the brain^101^ and is expressed in plasma and cerebrospinal fluid in proportion to adipocyte size^102^ and adiposity^103^, suggesting that leptin conveys energy sufficiency. However, individuals with obesity have elevated plasma leptin^100^, supporting leptin resistance as a comorbidity of obesity. Collectively, these studies showed NAc insulin promotes reward seeking by encoding reward salience via increased dopamine release and maintenance of dopamine reuptake, and, whereas food restriction may prime dopamine responses through insulin, HFD-induced insulin and leptin resistance may reduce NAc dopamine neurotransmission to alternately promote food seeking. A putative contributor to leptin and insulin resistance associated with diet-induced obesity is chronic inflammation triggered by saturated fats and rapid adipose tissue expansion^38,104,105^.

## HFD and obesity drive inflammatory processes that modulate dopamine control of food intake

Adipose tissue expansion in obesity reduces blood flow to adipocytes to induce hypoxia and release of cytokines causing local and systemic inflammation^104^. Indeed, increased expression of inflammatory genes triggered by hypoxia-inducible factor 1α were found in the adipose tissue of insulin-resistant individuals with obesity^106^. Further, obesity and HFD intake both stimulated cytokine release from peripheral and central immune cells^106–108^. Saturated fatty acids promote inflammation directly by promoting lipopolysaccharide (LPS) absorption^109^ and activating macrophages, microglia and astrocytes similarly to LPS by binding to toll-like receptor-4 (TLR4) and binding partners cluster of differentiation 14 and myeloid differentiation factor-2 (MD-2) to prompt receptor internalisation^38,39,108,110^. Toll-like immune receptors recognise pathogens, trigger nuclear factor-kappa B (NF-κB) signalling, and promote cytokine release^38,111^. Macrophages exposed to saturated fatty acids showed direct binding to MD-2 and TLR4, increased NF-κB and MAPK signalling, and interleukin-6 (IL-6) and tumour necrosis factor-α (TNFα) release^39^. Additionally, TNFα dampened the insulin signal via serine phosphorylation of insulin receptor substrate 1 in adipocytes^112^ whereas loss-of-function mutations in TNFα and TNFα receptors prevented HFD-induced insulin resistance^113^. Similarly, TLR4 gene mutation protected against HFD-induced obesity and promoted insulin signalling^105^. Insulin resistance due to inflammation is one key mediator of HFD-induced alterations to dopamine.

Inflammatory cytokines decrease dopamine packaging and signal transduction via reduced function and expression of vesicular monoamine transporter 2 and D2R but increase function or expression of DAT, which alters dopamine reuptake^114^. Additionally, inflammatory cytokines and reactive oxygen species reduced availability of the cofactor tetrahydrobiopterin required by TH for dopamine synthesis^115^. Further, systemic administration of proinflammatory cytokines IL-6 and IL-2 decreased NAc extracellular dopamine, though IL-1β had no effects^116^. Inflammatory processes may lower synaptic dopamine and alter feeding behaviours. Indeed, TLR4 knockout (KO) mice exhibited reduced preference for fat and sugar and attenuated WD-induced food intake, weight gain and palatable food preference^117^. Additionally, saturated HFD but not monounsaturated fat increased anxiety and depressive behaviour in conjunction with increased plasma cytokines and NAc cytokine and NF-κB transcriptional activity associated with heightened expression of microglial and astrocytic markers^118^. Further, intake of a free-choice cafeteria diet altered morphology of NAc MSNs and increased proinflammatory cytokine expression related to microglial activation, whereas microglial inhibition restored these effects and prevented diet-induced intake and weight gain^119^. Together, saturated fat and obesity may promote inflammation and insulin resistance that decrease dopamine synthesis, vesicular packaging, and capacity for dopamine release and reuptake. However, we posit that HFD-induced neuroinflammation uniquely promotes insulin resistance within the NAc as a primary driver of reduced reward value that promotes overconsumption of palatable foods, but a major gap involves lack of pharmacological investigation assessing interactions between insulin, LPS, inflammatory cytokines and microglial activation on behaviour and presynaptic dopamine neurotransmission in the NAc after chronic HFD intake during obesity.

## Neuroinflammation interacts with stress to modulate dopamine and food intake

Stress encompasses a variety of homeostatic disruptions which may be acute or chronic and physiological or psychological in nature. Food intake and body weight can change in response to stress depending on the type, intensity and duration of the stressor and activation of specific stress circuits. As discussed above, the physiological stress of diet-induced inflammation alters dopamine signalling in the NAc. Likewise, psychological stress related to substance use disorders^120^ and diet-induced^118,121^ anxiety have been linked to disruptions in dopamine homeostasis. This next section highlights how acute or chronic stress responses alter food intake, discussing the impacts of dietary fat and induction of inflammation on these processes.

### Acute stress: role of HPA, CRF and inflammation on food intake

**Fig. 2.**
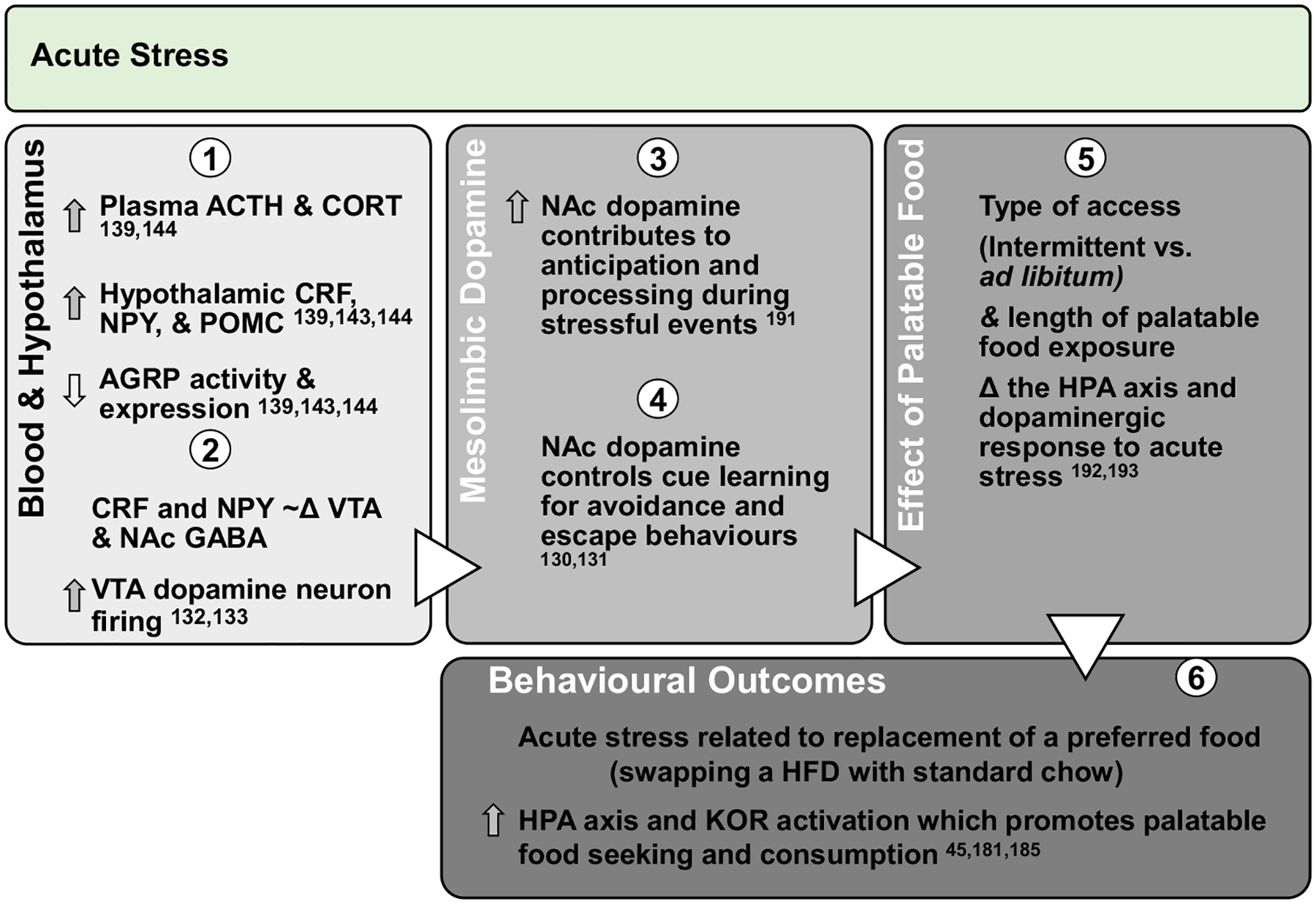
Effect of acute stress on dopamine neurotransmission and palatable food intake: acute exposures to stress engage the PVN and promote sympathetic and HPA axis activation leading to direct engagement of dopamine pathways. Downstream, glucocorticoid response and promotion of anorectic versus orexigenic neuropeptides inhibit food intake. However, acute stress associated with short-term removal of HFD activates stress systems that may persist for extended periods to promote seeking and intake of palatable foods. Further, activation of stress systems may reduce chow intake but promote consumption of palatable foods that acutely activate NAc dopamine neurotransmission and provide an alternate ‘avoidance or escape’ behaviour. ACTH, adrenocorticotropin; CORT, corticosterone/cortisol; CRF, corticotrophin-releasing factor; NPY, neuropeptide Y; POMC, pro-opiomelanocortin; AGRP, agouti-related peptide; HPA, hypothalamic–pituitary–adrenal; KOR, κ-opioid receptor; Δ, change.

Acute stress encompasses a huge variety of physiological and psychological triggers that activate the hypothalamic–pituitary–adrenal (HPA) stress axis in a coordinated effort with metabolic, immune, autonomic nervous, and digestive systems to increase breathing and heart rate but slow digestion in preparation for ‘fight or flight’ response^122,123^. Various stressors induce the hypothalamic paraventricular nucleus (PVN) to release corticotrophin-releasing factor (CRF), stimulating the pituitary gland to release adrenocorticotropin (ACTH) that targets adrenal glands to release glucocorticoids (corticosterone in rodents) and promote stress responses^124^. The hypothalamus also activates sympathetic neurons in response to stress that induce adrenal release of epinephrine and NPY to mobilise nutrients via glycogenolysis and gluconeogenesis^124^. In this context, CRF acutely produces anorexia and weight loss that may last several days^124^. Indeed, either a single exposure to LPS or acute immobilisation stress reduced food intake and body weight that persisted for over a week^125^. Human studies support this construct, as the most stressful event of each day increased the likelihood of eating less^126^, which was mediated by delayed digestive processes induced by acute stress^127^. Acute stress in healthy adults lacking comorbidities and stressors related to obesity appear to slow digestion and reduce food intake. However, induction of inflammation during saturated HFD consumption could interact with classical stress systems to promote effects of stress on reward pathways. For example, intraperitoneal LPS injection increased plasma ACTH and corticosterone and hypothalamic/pituitary proinflammatory cytokines in CRF KO and wild type mice^128^. Reciprocally, CRF promoted cortical microglial proliferation and dose-dependently increased TNFα release mediated by phosphorylation of MAPK intracellular signalling proteins shared by TLR4 activation^129^. Therefore, HPA axis and proinflammatory molecules engage in intracellular crosstalk and can independently promote stress. Acutely, stress mobilises nutrients and dampens food intake, but these behavioural effects might be altered during up-regulated inflammatory signalling. Overall, effects of HFD intake on the interactions between inflammatory and stress systems in the NAc have not been tested to determine effects on dopamine or behaviour during obesity or diet-related stressors (Fig. 2).

The immediate effects of acute stress are contextual and influenced by environmental factors. Interestingly, NAc dopamine release was increased during cues predicting foot shock, decreased during foot shock, then stimulated after lever pressing to stop the shock^130,131^. This shows NAc dopamine neurotransmission is involved in learning behaviours to avoid negative stimuli. Stress may directly induce dopamine activity, as CRF dose-dependently increased VTA dopamine neuron firing which was abolished by antagonising CRF receptor 1^132^, and CRF may activate both VTA GABA and dopamine neurons^133^. However, pharmacological activation of the HPA axis increased progressive ratio response in HFD but not chow-fed groups, which was reversed by antagonising CRF receptor 1^134^. Importantly, acute stress does not occur in isolation, and up-regulation of inflammatory signalling by HFD consumption could alter effects of acute stress. For example, LPS application 24 h after acute, inescapable tail shock promoted hippocampal NF-κB, TNFα, IL-6 and IL-1β gene expression, corticosterone, and microglial NF-κB and IL-1β responsiveness that was fully attenuated when TLR2, TLR4^135^ and glucocorticoid receptors^136^ were blocked during stress, and this stress-induced priming of neuroinflammation was mediated by transition of microglia, but not astrocytes, from a quiescent to an active state^137^. There are also sex effects within stress-induced priming of inflammatory microglial activation. The same stress paradigm similarly primed central proinflammatory cytokines, reduced anti-inflammatory pathways, and acutely reduced sucrose intake in males and females^138^. However, LPS 24 h after tail shock or glucocorticoid injection induced peripheral proinflammatory cytokines and reduced central glucocorticoid receptors in females but induced microglial IL-6 and IL-1β mRNA expression specifically in males^138^. Overall, specific effects of acute stress on food intake depend on prior exposure to a stressor and complex peripheral and central inflammatory signalling, suggesting those with chronic, diet-induced elevated proinflammatory states might be primed to be more reactive and have alternate behavioural responses to chronic stress, though further study is required explore this interaction in dopaminergic centres that influence food intake, like the NAc.

### Chronic stress and food intake

Chronic, repeated stressors may affect food intake and body weight differently than acute stress, particularly in an obesogenic state which may promote socio-behavioural and physiological stressors. Effects of stress on hypothalamic nuclei may override homeostatic feeding. For example, inescapable foot shock engaged the HPA axis in addition to increased NPY but decreased AGRP expression, and α-melanocyte stimulating hormone released by ARC POMC/CART neurons increased stress-induced HPA activation^139^. Furthermore, associating a place or flavour with NPY/AGRP neuron activation reduced preference for that place or food^140^, and AGRP neuron activity was reduced due to food cues^140^ and initiation of feeding^141^. Therefore, negative valence associated with firing of AGRP neurons may provide motivation to perform behaviours that induce positive valence like eating^2^ which could provide a stress alleviating effect. This is important given obesity-prone rats fed HFD for 6 weeks had greater HPA axis activity with greater central CRF concentrations and plasma corticosterone than chow counterparts^142^. Therefore, the transition in stress response that occurs over repeated stress exposures could be exacerbated by diet-induced obesity and greater stress reactivity. Acute restraint decreased ARC AGRP expression^143^ but increased hypothalamic POMC expression in conjunction with reduced food intake^144^. Conversely, chronic restraint increased LH AGRP and decreased ARC melanocortin 4 receptor^143^ that responds to POMC. This showed unique activation of hypothalamic anorectic versus orexigenic signalling, respectively, in response to acute versus chronic stress. Diet-induced inflammation may mediate these effects, as acute lipid infusion or palmitate exposure in AGRP/NPY neurons increased TNFα, proinflammatory cytokines and NPY expression^145^. Conversely, 8-week HFD consumption reduced TNFα and AGRP expression while 20-week consumption promoted TNFα in NPY and AGRP neurons^145^. This suggests palatable foods acutely induce hypothalamic inflammation in regions that innervate the NAc followed by up-regulation of protective mechanisms which are eventually overcome with extended HFD intake, and dietary responses to chronic stress likely depend on inflammatory status related to food composition and availability associated with responsivity of NPY/AGRP and POMC/CART neurons and NAc dopamine cue learning.

**Fig. 3.**
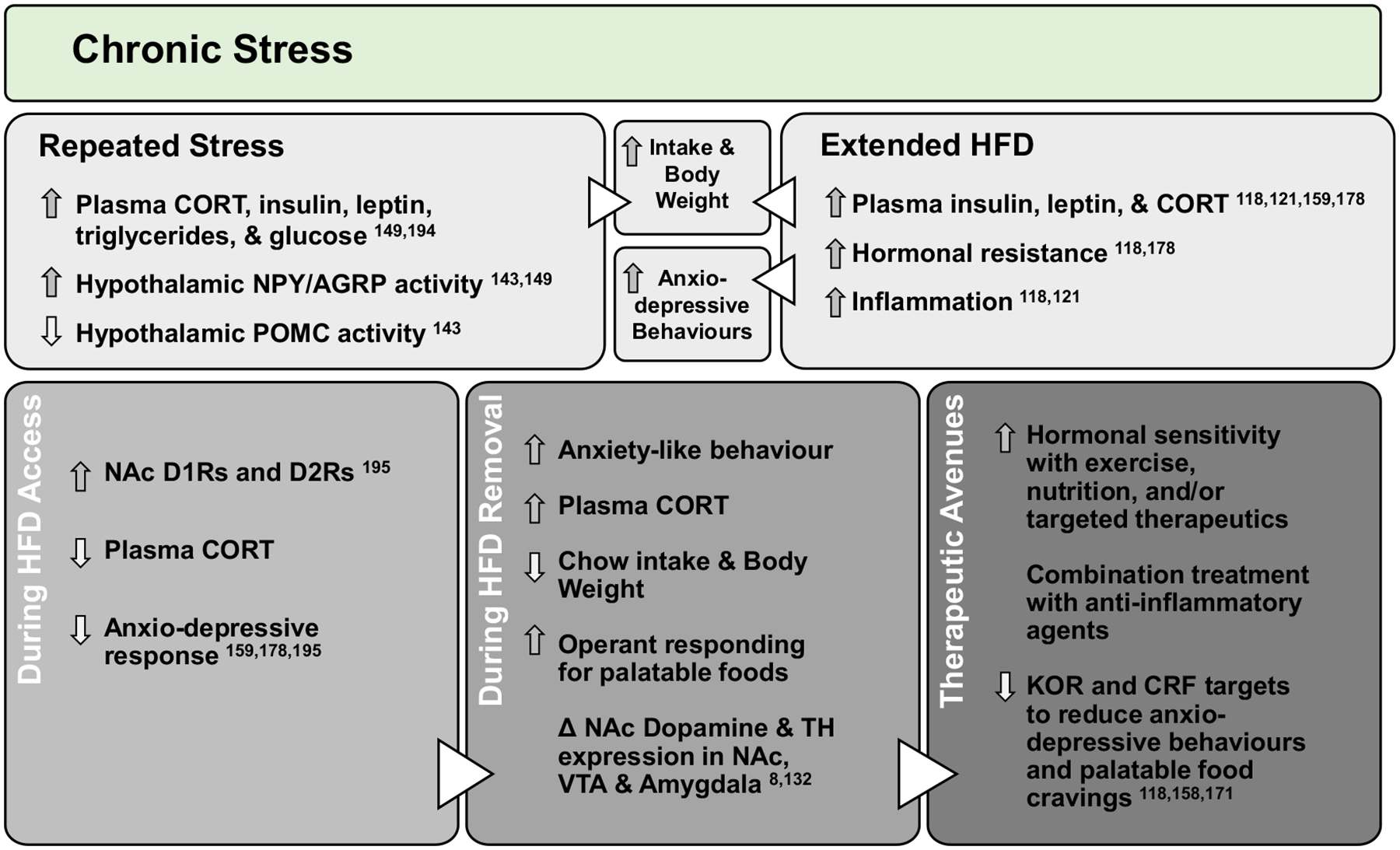
Chronic stress alters dopamine and promotes consumption of palatable food: repeated stress exposures, including long-term physiological stresses related to diet-induced obesity, chronically up-regulate stress pathways to promote orexigenic neuropeptides, inflammation and hormonal resistance. This leads to dysregulation of dopamine and increased food intake, weight gain and anxio-depressive behaviours. However, consumption of palatable foods during stress activates the dopamine system and reduces metabolic and behavioural responsivity to stress, highlighting stress alleviative properties of palatable foods. Behavioural and pharmacological interventions which improve diet-induced alterations to inflammatory, hormonal, stress and dopamine systems may reduce craving, seeking and consumption of highly palatable foods for obese individuals attempting to lose weight. CORT, corticosterone/cortisol; NPY, neuropeptide Y; AGRP, agouti-related peptide; POMC, pro-opiomelanocortin; D1R/D2R, dopamine type 1 or type 2 receptor; TH, tyrosine hydroxylase; KOR, κ-opioid receptor; CRF, corticotrophin-releasing factor; Δ, change.

Extended glucocorticoid responses may shift stress reactivity and impact hormonal regulation of energy stores. Glucocorticoids act to mobilise nutrients via gluconeogenesis^146^ and adipose tissue lipolysis^147^ during stress. However, these effects over an extended period could lead to hyperinsulinemia^147,148^, hyperleptinemia^149^, and insulin^148,150^ and leptin resistance. Indeed, 7 d of artificial glucocorticoid administration in healthy women increased food intake, plasma insulin and plasma leptin^151^, while exercise decreased cortisol response to stress (i.e. cortisol reactivity) accompanied by reduced disordered eating^152^. Maniam and Morris^153^ proposed glucocorticoid-induced stimulation of the dopamine system provides motivation to resolve stressful situations which may be ‘hijacked’ into motivation to obtain palatable foods. For example, in samples of women, presence of obesity increased cortisol reactivity^154^ which was associated with increased calorie consumption^155^. Downstream, glucocorticoids promote feeding by negatively regulating CRF and activating orexigenic NPY neurons^149,153^ that are inhibited by insulin and leptin and interconnected with the LH, NAc and VTA^2^. Therefore, during diet-induced obesity, chronic stress promotes glucocorticoid-induced activation of feeding centres and removes the stop signal from hypothalamus to VTA to promote dopamine-motivated feeding. It is important to note that glucocorticoids are involved in normal feeding responses, as restoring corticosterone to adrenalectomised rats increased saccharin^156^ and sucrose^157^ intake to control levels. On the other hand, access to lard blunted corticosterone response during restraint stress^158^. Therefore, diet interacts with glucocorticoids to modulate stress-induced feeding. Indeed, mice exposed to chronic social stress after 12 weeks on a HFD showed improved weight loss, reduced plasma insulin and leptin, and dampened anxio-depressive behaviours versus stressed controls or non-stressed HFD groups^159^, suggesting a potentially stress-alleviating effect of palatable food intake. Overall, diet-induced obesity produces neurochemical shifts and maladaptive stress responses, but an opportunity for future investigation lies in whether HPA axis and κ-opioid receptor stress responses drive food intake for obese individuals during the chronic diet-related stress of restricting calories for weight loss (Fig. 3).

### Effects of a HFD on κ-opioid-receptor-mediated stress, dopamine, and food intake

The HPA axis interacts with κ-opioid receptors (KORs) to gate NAc dopamine signalling. Indeed, expression of CRF, ACTH and glucocorticoids systemically and centrally are co-regulated with dynorphin, a ligand for KORs, and its precursor prodynorphin with ligands in either system inducing expression and release reciprocally to promote stress responses^160–163^. KORs are G-protein-coupled receptors that inhibit adenylyl cyclase and cAMP activity^164,165^ and are co-expressed within NAc DAT-expressing neurons^166^. Blocking NAc KORs inhibited dopamine reuptake and increased extracellular dopamine^167^, while KOR activation increased reuptake to limit extracellular dopamine^168,169^. KORs are present within NAc presynaptic dopamine terminals, MSNs, and GABAergic and cholinergic interneurons and reduce extracellular dopamine to promote feeding cessation^170^. However, ventricular KOR agonism promoted HFD intake during satiation, whereas KOR antagonism in a fasted state reduced HFD intake^171^, and systemic KOR agonism reduced NAc core phasic dopamine release parallel to reduced motivation to obtain sucrose^172^. This suggests KORs control extracellular dopamine to alter rewarding effects of food. In the NAc, KOR activation during nicotine exposure^173^ and amphetamine withdrawal^174^ decreased basal and evoked dopamine, while KOR antagonism attenuated alcohol self-administration during withdrawal^175^. Furthermore, acute stress promoted TNFα in the amygdala, and amygdalar TNFα potentiated anxiety during withdrawal that was blocked by CRF antagonism^176^. Therefore, HPA axis, KOR and inflammatory systems interact to induce dysphoria and relapse during withdrawal, and up-regulation of these symptoms during diet-induced obesity could promote food cravings when energy is restricted for weight loss.

Neuroinflammation interacts with stress and reward systems. Indeed, exposing women to stress induced plasma IL-6 concentrations that correlated with reduced NAc activation during an fMRI reward learning task^177^. Further, chronic intake of saturated HFD versus low fat^118,121,178^ or monounsaturated fat^118^ increased anxio-depressive behaviours, plasma corticosterone, and hypothalamic and NAc inflammatory cytokine and TLR4 expression. Conversely, prodynorphin overexpression promoted anti-inflammatory M2 versus M1 phenotype of hippocampal microglia and inhibited LPS-induced TLR4 activation of NF-κB to promote IL-4 and IL-10 but inhibit IL-1β and IL-6 release^179^. Reciprocally, intraplantar injection of IL-1β increased KOR mRNA expression in basal root ganglia neurons^180^. However, dynorphin, enkephalin and KOR expression in the NAc were not affected by WD intake or 18-d replacement with chow^181^ or 18 d of *ad libitum* or intermittent access to a HFD^182^. Therefore, while HFD intake itself does not appear to alter dynorphin/KOR expression, it appears that KORs dampen proinflammatory responses induced by saturated fat and could engage in intracellular crosstalk with inflammatory molecules similarly to the HPA axis. Human neuroimaging studies linked a dopaminergic response to the consumption of palatable food^183^, palatable food cues^29^ or smelling food odours^184^ with increased reports of pleasure^183,184^ and wanting^29^ of food. Therefore, changing a lifetime of food preferences by restricting energy intake to lose weight may be particularly stressful and activate stress pathways that dampen dopamine signalling.

In support of KOR system involvement during palatable food restriction, pair feeding of WD to chow intake levels in controls reduced NAc KOR expression in absence of obesity, but 18 d of WD replacement with chow did not alter KOR expression^181^. Therefore, it is possible that differences in KOR expression become neutralised over 18 d of diet replacement or by diet-induced weight gain, or that specifically KOR function could be up-regulated. HFD intake alters food preferences, as replacement of a WD^45^ or HFD^8^ with chow persistently reduced food intake and acutely increased plasma corticosterone and anxiety-like behaviour^8^. Further, whereas HFD intake promoted sucrose anhedonia^8,48,185^, palatable food removal increased preference for sucrose^48,185^ and motivation to obtain sucrose^8,45^ and fat^8^ that occurred as soon 3 d^8^ after diet removal but persisted for several weeks^45,48,185^. This indicates prolonged enhancement of salience for palatable food when a preferred diet is not available, and it is possible that KORs, which contribute to the rewarding properties of food, promote this effect. Further, HFD intake started at parturition reduced VTA TH and NAc D1R and D2R expression in male and female mice, but 4 weeks of HFD replacement exacerbated this reduction in the VTA of females and NAc of males but oppositely restored these effects in the VTA of males and NAc of females^48^. However, when HFD is introduced in adolescence, HFD replacement does not recover D2R expression in females with strong promotion of palatable food intake, though effects were attenuated during adult-onset HFD^185^. This suggests prolonged effects of a HFD on dopamine synthesis and signalling in the VTA alter the capacity for neurotransmission in the NAc. Changes in NAc neurotransmission occur via alterations to D1Rs and D2Rs resulting in increased salience of palatable foods when they are no longer available. These effects were significantly mediated by sex and age of palatable food introduction with high vulnerability during adolescence. Overall, individuals with clinical obesity or that persistently consume a HFD may be poorly adapted to control palatable food cravings during energy restriction owing to diet-induced or psychological stress that engages the HPA or KOR systems.

## Conclusions

NAc dopamine neurotransmission is affected by physiological state and access to highly palatable foods that promote obesity, inflammation and hormonal resistance. Chronic engagement of the HPA axis and KOR stress systems by repeated exposures to daily life stressors interact with the inflammatory and hormonal systems disrupted during obesity. Ultimately, palatable food intake acutely increases dopamine release and reuptake, but extended HFD intake reduces the capacity for dopamine neurotransmission. These trends are related to the physiological consequences of obesity that together promote the vulnerability to overeat in order to promote synaptic dopamine to combat obesity-related dysphoria and reduction of dopamine tone. The effect of dietary fat on dopamine’s influence overfeeding could be exacerbated by stress due to dietary restriction or removal of preferred foods. However, pharmacologically targeting receptors that mediate stress in the brain, like KORs, or controlling diet-induced inflammation that engages in crosstalk with KOR system-mediated stress may improve the success of weight loss interventions by attenuating the impact of stress on the dopamine system. Moreover, the dampening of dopamine neurotransmission by long-term consumption of a HFD, specifically high in saturated fat, could be potentiated by inflammation acting on dopamine neurons, which could heighten stress responses that further attenuate dopamine signalling. The result could be an increase in palatable food seeking and consumption to curb potential negative affect with an acute but transient increase in dopaminergic signalling.
